# Prognosis of severe acquired brain injury: Short and long-term outcome determinants and their potential clinical relevance after rehabilitation. A comprehensive approach to analyze cohort studies

**DOI:** 10.1371/journal.pone.0216507

**Published:** 2019-09-26

**Authors:** Bernardo Lanzillo, Giuseppe Piscosquito, Laura Marcuccio, Anna Lanzillo, Dino Franco Vitale

**Affiliations:** 1 Istituti Clinici Maugeri, IRCCS di Telese Terme, Via Bagni Vecchi 1, Telese T, BN, Italy; 2 Casa di Cura San Michele, Via Montella 16, Maddaloni, CE, Italy; Appalachian State University, UNITED STATES

## Abstract

**Background:**

Accurate prognostic evaluation is a key factor in the clinical management of patients affected by severe acute brain injury (ABI) and helps planning focused therapies, better caregiver’s support and allocation of resources. Aim of the study was to assess factors independently associated with both the short and long-term outcomes after rehabilitation in patients affected by ABI in the setting of a single Rehabilitation Unit specifically allocated to these patients.

**Methods and findings:**

In all patients (567) with age ≥ 18 years discharged from the Unit in the period 2006/2015 demographic, etiologic, comorbidity indicators, and descriptors of the disability burden (at hospital admission and discharge) were evaluated as potential prognostic factors of both short-term (4 classes of disability status at discharge) and long-term (mortality) outcomes. A comprehensive analytical method was adopted to combine several tasks. Select the factors with a significant independent association with the outcome, assess the relative weights and the “stability” (by bootstrap resampling) of them and estimate the role of the prognostic models in the clinical framework considering “cost” and “benefits”. The generalized ordered logistic model for ordinal dependent variables was used for the short-term outcome while the Cox proportional hazard model was used for the long-term outcome. The final short-term model identified 7 factors that independently account for 37% of the outcome variability as shown by pseudo R^2^ (pR^2^) = 0.37. The disability status descriptors show the strongest association since they account for more than 60% of the pR^2^, followed by age (14.8%), the presence of percutaneous endoscopic gastrostomy or nasogastric intubation (14.4%), a longer stay in the acute ward (5.9%) and concomitant coronary disease (1.3%). The final multivariable Cox model identified 4 factors that independently account for 52% of the outcome variability (R^2^ = 0.52). The disability extent and the disability recovered lead the long-term mortality since they account for the 53% of the global R^2^. The relevant effect of age (42%) is appreciable only after 2 years given the significant interaction with time. A longer stay in the acute ward explains the remaining fraction (5%). Considering ‘cost and benefits’, the decision curve analysis shows that the clinical benefit achieved by using both prognostic models is greater than the other possible action strategies, namely ‘treat all’ and ‘treat none. Several less obvious characteristics of the prognostic models are appreciated by integrating the results of multiple analytical methods.

**Conclusion:**

The comprehensive analytical tool aimed to integrate statistical significance, weight, “stability” and clinical “net” benefit, gives back a prognostic framework explaining a relevant portion of both outcomes’ variability in which the strong association of the disability status with both outcomes is comparable to and followed by a time modulated role of age. Our data do not support a differentiated association of traumatic vs non-traumatic etiology. The results encourage the use of integrated approach to analyze cohort data.

## Introduction

Severe acquired brain injury (ABI) describes cerebral damage of different etiologies. ABI is characterized by the risk of varying degrees of serious cognitive and physical impairments, and places a burden on society due to its, often-devastating, impact on health, on the lives of the patient’s relatives, and on the economy.

The development of efficient prognostic tools for every stage of the complex clinical course of ABI would enable planning of focused therapies, allocation of resources, and assist caregivers to provide the best support for patients.

The etiology of ABI is wide-ranging, from traumatic brain injury to vascular (ischemic or hemorrhagic), anoxic, neoplastic, inflammatory or metabolic causes. However, given the high incidence of traumatic causes (prevalence 40–59%), most reports have focused on this etiology, with occasional information on other causes being treated as a whole (as non-traumatic brain injury) [1–4].

There is insufficient information on factors related to short- and long-term outcomes in patients with ABI and, given the high prevalence of traumatic causes, most studies concern only trauma-specific aspects [5–8] or compare traumatic ABI with ABI of hemorrhagic and anoxic etiologies [9]. Moreover, the current literature is often limited to univariate analysis of factor association and does not consider confounding factors in the populations under examination [10–11].

The aim of this study was to investigate factors associated with short and long-term outcome following rehabilitation in a group of subjects with ABI of various etiologies, who were admitted to rehabilitation after the acute phase of the condition.

Multivariable analysis was used to adjust for confounding in evaluation of the weighting of each factor. In the effort to obtain an evaluation of the outcome-factors relationship as accurate (and detailed) as possible a coherent procedure was adopted (see Supporting Information) aiming to have an estimate of factor’s weight, random variability of the results and clinical relevance. The potential clinical relevance of the multivariable prognostic model was estimated by balancing the "costs and benefits" of applying the model in the same study population.

The short-term outcome was measured as the degree of disability (from none to death), at discharge, of a population of patients who were admitted for rehabilitation to a single institution ward dedicated to the treatment of patients with severe ABI of various etiologies. The incidence of death in the patients discharged alive identifies the long-term outcome.

## Material and methods

### Study population

Study data were gathered from the Intensive Neurorehabilitation Unit, established on 1 June 2006 as a specialized ward dedicated to patients with ABI, of the Istituti Clinici Maugeri, Telese Terme, Italy. All patients admitted to the rehabilitation unit had had brain injury with a Glasgow Coma Scale (GCS) score ≤8 for a period of at least 24 hours in the acute stage. On admission to the unit patients underwent a systematic protocol. Electronic data collection included both clinical management and epidemiologic observations. All patients admitted to the rehabilitation unit from 1 June 2006 to 31 December 2015 (*n* = 605) were included in the study. Exclusion criteria were: age less than 18 years (*n* = 30), voluntary rehabilitation hospital discharge (*n* = 5), and rehabilitation hospital discharge due to acute illness within 3 days of admission (*n* = 3). Thus, the final study population for the short-term outcome comprised 567 patients. All patients discharged alive (458) from the rehabilitation unit were checked for survival on 1st March 2016 by inspecting the national death certificate repository. One of the patients was not resident in Italy and was lost to follow-up after rehabilitation hospital discharge. The final study population for the long-term outcome, therefore, comprised 457 subjects.

All patients gave their informed written consent to participate to the study. In case the patient was unable to consent, the closest relative was informed and asked to give written consent. The study protocol was approved on February 27, 2019 protocol n° 11/18 OSS by the Institutional Ethics Committee: Istituto Nazionale per lo Studio e la Cura dei Tumori "Fondazione Giovanni Pascale"—Napoli Comitato Etico IRCCS Pascale.

Several factors registered at admission and at discharge of the rehabilitation hospital were considered potential predictors of discharge and long-term outcome respectively. These factors represent a range of attributes: basic demographic characteristics (age, sex), presence of comorbidity (hypertension, diabetes, chronic obstructive pulmonary disease (COPD), chronic kidney disease (CKD), coronary artery disease (CAD) and active infectious disease (AID)), interval between onset of the acute event and admission to the rehabilitation setting (OAI), nutritional status, presence of pressure ulcers at hospital admission, percutaneous endoscopic gastrostomy or nasogastric intubation (PEG/NGI), and tracheostomy. CKD was defined as a glomerular filtration rate ≤60 ml/min, measured with the Chronic Kidney Disease Epidemiology Collaboration equation. Six etiologies were identified in our study population: anoxia, bleeding, neoplasm, hypoxia, ischemia, and trauma. The length of the rehabilitation hospital stay (LOS) was considered for potential association with long-term outcome.

Five indices descriptive of the extent of disability were measured at rehabilitation admission, the Disability Rating Scale (DRS), the Level of Cognitive Functioning Scale (LCFS), the Extended Glasgow Outcome Scale (GOSE), and both the cognitive and motor domains of the Functional Independence Measure (FIM^TM^) score. The same measures were repeated at rehabilitation discharge and the amount of disability gained (or lost) during rehabilitation hospital stay, measured as the variation between rehabilitation discharge/admission normalized as the percent gain of the maximum possible recovery attainable evaluated at rehabilitation admission, was computed for all 5 indices [12].

The severity of disability at rehabilitation hospital discharge was taken as the short-term outcome. This was evaluated by collapsing the DRS discharge assessment score into four classes: class I if no or mild disability (DRS 0–1) was observed, class II for partial to moderately severe disability (DRS 2–11), class III for severe disability to extreme vegetative state (DRS 12–29), and class IV for in-hospital death (DRS 30).

### Statistical analysis

Continuous factors are expressed as means±standard deviations (SD), while categorical factors are expressed as percent and number of occurrences. Since univariate analysis does not assess the association of each factor independent of the effect of other factors, a multivariable procedure specific to each outcome (short and long-term) was used to analyze both the relationship between rehabilitation hospital admission factors and short-term outcome probabilities and the relationship between rehabilitation hospital discharge and long-term outcome probabilities (see details of the model building strategy in the S1 Appendix).

Briefly, the generalized ordered logit (GOLOGIT) model for ordinal dependent variables [13] was used to model the independent association of each factor with the probability of each class of the short-term outcome, while the Cox proportional hazard analysis was used to identify and measure the independent contribution of each factor associated with all-cause mortality.

Significant variables were selected from a pool of 19 and 15 candidates for the short and long-term outcome respectively. The criteria adopted for pool definition are detailed in the S1 Appendix.

Graphs of the probability of each class of the short-term outcome were plotted relative to a clinically meaningful interval of values for the continuous or categorical factors that were found significant in the multivariable analysis and covered a span inside the range observed for each variable. However, plot interpretation should take into account that interpolation at the range borders is prone to a greater variability. Probabilities were derived from the GOLOGIT model coefficients and “population adjusted” for the confounding effect of the other significant factors. This method results in estimates of the probabilities that would occur if all subjects in the study population had the factor of interest at the chosen value. This enables visual assessment of the intrinsic effect of each factor independent of the other factors.

Similarly, the "population adjusted" method was used to obtain a graphical representation of the impact of each significant factor on mortality. Each population adjusted curve, estimated at a specific factor value, was compared to the overall observed mortality incidence curve, and exemplifies the mortality that would be observed if all patients in the study population had the given specific factor value.

The adjusting procedure was suggested by Nieto [14] and has been applied by others [15–16] to adjust survival curves for confounding. As detailed in the S1 Appendix, the method resides on averaging the estimate obtained for each subject in the population studied (the GOLOGIT derived probabilities for the short-term model and the Cox estimated survival curve for the log-term model). If the factor values used to fit the model are that observed in each subject these average estimates would result in the observed 4 outcome class frequencies (GOLOGIT model) and in the overall Kaplan-Meyer survival curve (Cox model). Employing specific factor values results in estimating the effect that this factor change would have on the outcome considered.

For both the Cox and the GOLOGIT models the functional form of the association between continuous factors and outcome was checked and modelled using a multivariable fractional polynomial (MFP) algorithm [17], which, in case of the GOLOGIT model, adds to the non-linearity/linearity fit inherent in the partial proportional odds model option used by this procedure (see S1 Appendix).

The relative weight of each significant factor was estimated by measuring the partial contribution of each variable to the variation in global outcome, as measured by global pseudo R^2^ (pR^2^) in the GOLOGIT model and by the global explained variance (R^2^) in the Cox model, using the Shapley-Owen decomposition algorithm [18].

The discrimination ability of the final prognostic GOLOGIT model was estimated by the area under the curve (AUC) of the receiver operating characteristics (ROC) analysis for each of the four outcomes, as shown in S1 Fig.

The stability (internal validity) of the results obtained with both Cox and GOLOGIT final models was measured by the frequency that each factor was selected as "significant" in a large series (1000) of bootstrap replications of the dataset by applying the same procedure [19]. Similarly, the stability of the selected functional form was measured by the frequency of significant linear vs non-linear association in the same collection of bootstrap replications.

The model building strategy of the Cox model included the verification of the proportional assumption of the model and, if necessary, the adoption of an extended model with the MFPT algorithm [20].

In order to evaluate the potential impact of the final prognostic model on the clinical environment, considering "costs and benefits", decision curve analysis (DCA) was employed [21]. DCA computes the net benefit obtained by applying the investigated prognostic model to make a clinical decision. In this framework “make a clinical decision” refers to a “non-specific clinical action” by which patients are “treated” with the aim to improve their prognosis. As detailed in the S1 Appendix, a net benefit curve is obtained by “treating” or not “treating” using the criteria suggested by the investigated prognostic model. This curve is then compared to that obtained applying the clinical action to all subjects irrespective of the prognostic tool (“treat all”) and to that obtained not applying the clinical action to any of the subjects (“treat none”). All curves (“treat by prognostic model”, “treat all” and “treat none”) are taken along the full observed range of the “decision threshold”, i.e. the threshold where the “advantages” of being treated with the “clinical action” being a “case” equal the “disadvantages” of being treated with the “clinical action” not-being a “case”.

For each of the four outcome classes of the GOLOGIT model the net clinical benefit of using the final prognostic model was compared to the "treat all" and "treat none" criteria. To avoid the "optimistic bias" inherent in computation of individual outcome probabilities, the net benefit, as well as the ROC curves, were computed using the jack-knife procedure (i.e. the outcome probabilities for a given patient, entered into DCA and ROC analysis, were estimated using a model that was built excluding the subject under assessment). Similarly, the potential usefulness of the final prognostic Cox model in the clinical decision-making process was evaluated by an extension to censored data of the DCA analysis [22]

Univariate comparisons between the four short-term outcome classes or between long-term survivors and non survivors were performed using analysis of variance (ANOVA) or χ^2^ test for continuous and categorical factors, respectively. Kruskal–Wallis test was used in the case of not normally distributed variables by Shapiro–Wilk test.

The study was conducted according to the Strengthening the Reporting of Observational Studies in Epidemiology (STROBE) statement [23].

Data were analyzed using Stata version 15.0 (StataCorp LP, College Station, TX, USA). Statistical significance was accepted at *p*≤0.05 for univariate comparisons and for variable selection, as well as for testing between variable transformations within the MFP procedure.

## Results

### Demographic and clinical characteristics of patients

Tables 1 and 2 show the characteristics at rehabilitation hospital admission and discharge respectively.

**Table 1:** Characteristics of the study population stratified by outcome classes.

	All	Outcome class I	Outcome class II	Outcome class III	Outcome class IV	p value
	(n = 567)	4.9% (28)	43.2% (245)	32.6% (185)	19.2% (109)	
**Age, yrs (mean**±SD)	55.1±17.7	39.8±16.3	48.9±17.4	59.4±15.5	65.6±13.9	≤0.0001
**Gender, % Male (n)**	63.5% (382)	75.0% (21)	66.5% (163)	57.8% (107)	63.3% (69)	0.2
**Hypertension, % (n)**	39.7% (225)	17.9% (5)	39.2% (96)	41.6% (77)	43.1% (47)	0.09
**Diabetes, % (n)**	13.6% (77)	7.1% (2)	10.6%(26)	15.7%(29)	18.4% (20)	0.1
**COPD, % (n)**	9.4% (53)	0.0% (0)	6.9% (17)	10.8% (20)	14.7%(16)	0.03
**GFR <60 ml/mg, % (n)**	8.8% (50)	3.6% (1)	6.9%(17)	8.1% (17)	15.6%(15)	0.12
**CAD, % (n)**	10.9% (62)	14.3% (4)	7.7% (19)	10.3% (19)	18.4% (20)	0.04
**AID, % (n)**	38.6% (219)	28.6% (8)	25.3% (62)	50.8% (94)	50.5% (55)	≤0.0001
**OAI, wks**	8.3±6.9	5.5±3.0	6.5±3.7	10.1±9.6	9.7±6.8	≤0.0001
**NI, % (n)**	8.5% (48)	10.7% (3)	7.4%(18)	8.1% (15)	11.0%(12)	0.8
**SPU, % (n)**	39.5% (224)	14.3%(4)	23.7% (58)	54.1% (100)	56.9%(62)	≤0.0001
**PEG/NGI, % (n)**	57.0% (323)	14.3% (4)	27.8% (68)	85.4% (158)	85.3% (93)	≤0.0001
**Tracheostomy, % (n)**	63.0% (357)	35.7% (10)	42.0% (103)	80.5% (149)	87.2% (95)	≤0.0001
**Aetiology**						≤0.0001
**Anoxia, % (n)**	14.6% (83)	7.1% (2)	4.9% (12)	22.2% (41)	25.7% (28)	
**Bleeding, % (n)**	34.0% (193)	32.1% (9)	33.9% (83)	37.3% (69)	29.4% (32)	
**Infection, % (n)**	8.3% (47)	14.3% (4)	9.4% (23)	6.5% (12)	7.3% (8)	
**Hypoxia, % (n)**	5.3%(30)	10.7% (3)	8.6% (21)	1.6% (3)	2.8% (3)	
**Ischemia, % (n)**	12.7% (72)	3.6% (1)	9.8% (24)	12.4% (23)	22.0% (24)	
**Trauma, % (n)**	25.0% (142)	32.1% (9)	33.5% (82)	20.0% (37)	12.8% (14)	
**DRS score (mean±SD)**	19.6±5.5	12.9±4.1	16.3±4.4	22.6±3.6	23.9±4.0	≤0.0001
**LCFS score (mean**±SD)	3.9±2.1	6.3±1.7	5.1±1.6	2.9±1.5	2.5±1.8	≤0.0001
**GOSE score (mean**±SD)	2.9±0.9	3.8±0.9	3.3±0.9	2.5±0.5	2.3±0.5	≤0.0001
**FIM** **motor score(mean**±SD)	17.0±8.8	25.8±12.8	19.7±10.7	13.9±4.2	13.8±4.3	≤0.0001
**FIM** **cognitive score (mean**±SD)	12.3±9.0	23.4±9.0	16.8±8.9	7.5±5.4	7.4±5.6	≤0.0001

SD = Standard Deviation. COPD = chronic obstructive pulmonary disease. GFR = glomerular filtration rate. CAD = coronary artery disease. AID = active infection disease. OAI = acute onset to admission interval. LOS = length of stay. NI = nutritional impairment. SPU = skin pressure ulcers. PEG/GI = percutaneous endoscopic gastrostomy or nasogastric intubation. DRS = Disability Rating Scale. LCFS = Level of Cognitive Functioning Scale. FIM = Functional Independence Measure .GOSE:Extended Glasgow Outcome Scale. P value refer to ANOVA/Shapiro Wilks analysis for continuous variables and to chi2 test for categorical factors.

**Table 2:** Discharged population characteristics.

	All	Survivors	Non Survivors	p value
	(n = 457)	(n = 350)	(n = 107)	
*Age yrs	52.6±17.6	49.7±17.8	61.9±13.6	<0.0001
*Gender (male), % (n)	63.5 (290)	64.3 (225)	60.8 (65)	= 0.51
*Etiology				= 0.005
**Anoxia, % (n)**	12.0 (55)	9.1 (32)	21.5 (23)	
**Bleeding, % (n)**	35.2 (161)	34.3 (120)	38.3 (41)	
**Infection, % (n)**	8.5 (39)	9.1 (32)	6.5 (7)	
**Hypoxia, % (n)**	5.9 (27)	6.3 (22)	4.7 (5)	
**Ischemia, % (n)**	10.5 (48)	10.3 (36)	11.2 (12)	
**Trauma, % (n)**	27.8 (127)	30.9 (108)	17.8 (19)	
*SPU, % (n)	18.6 (85)	41.2 (35)	58.8 (50)	<0.0001
*NI, % (n)	7.9 (36)	7.7 (27)	8.4 (9)	= 0.82
*AID, % (n)	35.7 (163)	30.9 (108)	51.4 (55)	<0.0001
*Diabetes, % (n)	12.4 (57)	10.3 (36)	19.6 (21)	= 0.01
*GFR<50 ml/mg, % (n)	7.2 (33)	6.9 (24)	8.4 (9)	= 0.59
*Tracheostomy, % (n)	26.9 (123)	18.6 (65)	54.2 (58)	<0.0001
*CAD, % (n)	9.2 (42)	9.7 (34)	7.5 (8)	= 0.48
*COPD, % (n)	7.9 (36)	6.9 (24)	11.2 (12)	= 0.14
*LOS, d	142.8±120.3	124.3±101.2	203.2±154.2	<0.0001
*OAI (1/x2), wks	7.9±6.9	7.4±6.0	9.6±9.0	= 0.02
*Discharge DRS	11.3±7.9	9.1±6.8	18.6±6.9	<0.0001
**Discharge LCFS**	6.1±2.3	6.7±1.9	4.2±2.5	<0.0001
**Discharge GOSE**	4.5±2.0	5.0±1.9	2.9±1.3	<0.0001
**Discharge cognitive FIM**	21.5±11.0	24.1±9.9	12.9±10.1	<0.0001
**Discharge motor FIM**	45.2±28.1	52.0±27.1	23.0±18.4	<0.0001
**In-hospital Gained Rescue**				
*DRS gain, %	45.3±31.2	53.8±28.1	17.8±27.3	<0.0001
**LCFS gain,%**	67.3±39.2	76.2±34.4	38.1±39.9	<0.0001
**GOSE gain, %**	42.9±30.1	49.9±29.1	20.1±20.7	<0.0001
**Cognitive FIM** **gain, %**	46.5±35.0	53.9±33.1	22.1±29.9	<0.0001
**Motor FIM** **gain**^**, %**^	39.9±35.0	48.0±34.0	13.2±22.7	

SPU: skin pressure ulcers. NI: nutritional impairment. AID: acute infectious disease. GFR: glomerular filtration rate. CAD: coronary artery disease. COPD: chronic obstructive pulmonary disease. LOS: length of hospital stay. OAI: acute onset to admission interval. DRS: Disability Rating Scale. LCFS: Level of Cognitive Functioning Scale. FIM: Functional Independence Measure. GOSE: Extended Glasgow Outcome Scale.

*Factors tested in the Cox analysis.

The characteristics of the study population at rehabilitation hospital admission, as a whole and stratified by short-term outcome class, are shown in Table 1. Table 2 shows the characteristics of the patients discharged alive and grouped by the long-term outcome.

At rehabilitation admission, the most frequent etiology was bleeding (34.0%), followed by trauma (25.0%), anoxia (14.7%), ischemia (12.7%), neoplasia (8.3%), and hypoxia (5.3%). At rehabilitation admission 37.6% of patients (*n* = 213) were in a vegetative state, 24.0% (*n* = 136) were in a minimally consciousness state, and the remaining 38.4% (*n* = 218) were conscious. Univariate analysis found statistically significant differences between the 4 outcome groups for 13 of the 20 factors considered. Similarly, among patients discharged alive, the group of long-term survivors had statistically significant differences in 10 of the 15 characteristics tested.

However, since univariate comparisons do not account for the confounding of each factor with others, we rely on the multivariable analyses (GOLOGIT and Cox) in order to draw conclusions about the association between factors and outcomes.

### Multivariable analysis of short-term outcome

Of the study population 4.9% were discharged from rehabilitation hospital with none-to-mild disability (outcome class I), 43.2% with partial-to-moderately-severe disability (outcome class II), 33.6% with severe-to-extreme disability (outcome class III), and 19.2% died in hospital (outcome class IV). The seven factors that, independently, are significantly associated with the outcomes are shown in Table 3 (full results are shown in S1 Table).

**Table 3:** Factors significantly associated with short-term outcome at the ordered multinomial logistic analysis.

Global pR^2^ = 0.37			
	pR^2^ contribution (%)	BIF (%)	Functional Form
Age	14.8	100.0	Lin
Event-Admission (wks)	5.9	99.3	Lin
Admission DRS	23.4	96.2	Lin
Admission LCFS	21.5	96.8	Non Lin
Admission GOSE	18.8	78.0	NA
PEG/NGI	14.4	83.7	NA
CAD	1.3	65.9	NA

DRS = Disability Rating Scale score. CAD = coronary artery disease. PEG/GI = percutaneous endoscopic gastrostomy or nasogastric intubation. GOSE = Extended Glasgow Outcome Scale. LCFS = Level of Cognitive Functioning Scale. pR^2^ = pseudo R^2^. BIF = Bootstrap Inclusion Frequency. Lin = Linear. NA = Not applicable.

The final GOLOGIT model has excellent goodness-of-fit (pR^2^ = 0.37), and good discrimination ability, as shown by the AUC of the four outcomes, which range from 0.82 to 0.88 in the ROC analysis. A non-significant (*p* = 0.24) Hosmer-Lemeshow goodness-of-fit test excluded mis-calibration. The calibration plot shows good agreement over the full range of probability (see S2 Fig).

The impact of each significant factor, as documented by its individual contribution to global pR^2^, includes a wide range of values. Rehabilitation admission DRS accounts for the greatest proportion (23.4%) of global pR^2^, followed by admission LCF and GOSE (21.5% and 18.8%, respectively). Age (14.8%) and PEG/NGI (14.4%) share a lower proportion of global pR^2^, while the interval between the acute event onset and admission to the rehabilitation setting (OAI) accounts for 5.9%. The presence of CAD exerts the smallest influence on global pR^2^ (1.3%).

**Fig 1:**
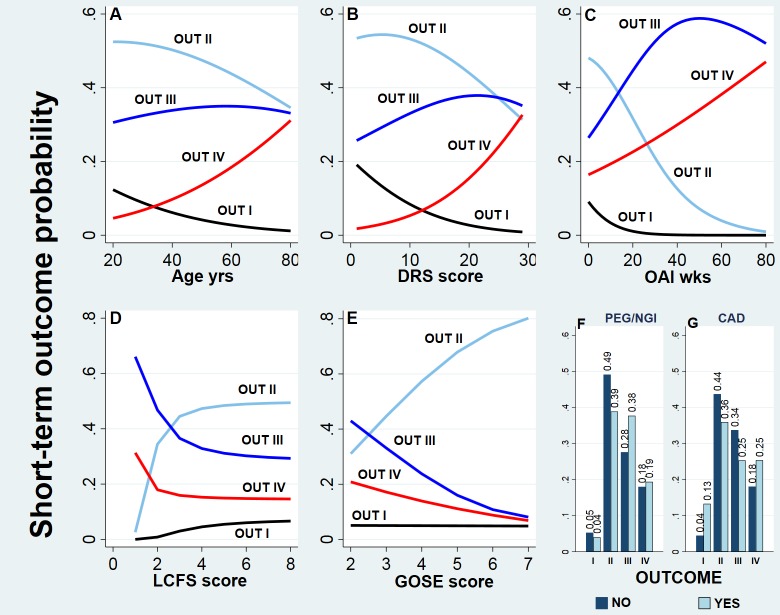
Short-term outcome probabilities. (A–E) Graphs of outcome probabilities (y-axis) relative to relevant intervals (x-axis) of the continuous factors age, Disability Rating Scale (DRS) score, acute event onset to hospital admission interval (OAI), Level of Cognitive Functioning Scale (LCFS) score, and Extended Glasgow Outcome Scale (GOSE). (F) Bar plots of the outcome probabilities of the presence/absence of percutaneous endoscopic gastrostomy/nasogastric intubation (PEG/NGI), and (G) coronary artery disease (CAD). OUT I to IV: short-term outcome classes I to IV.

Fig 1 shows the plots of the 4 outcome probabilities relative to meaningful intervals of continuous factors (selected within the observed range) and to the presence/absence of CAD and PEG/NGI.

Outcome probabilities over the span of each predictor (from Fig 1A to Fig 1G) were adjusted for confounding of the other significant factors in the final model, and estimate the probabilities that would be observed in the population if all patients had the given value of the specific predictor under consideration.

Increase in age correlates with a continuous reduction in the probability of outcome classes I and II, matched by an increase in the remaining two outcome classes.

Greater values of the DRS score measured at rehabilitation hospital admission, from 0 to 8–10, are associated with a reduction in outcome I probability, matched by an increase in outcome classes III and IV, with no noticeable changes in outcome class II. Further greater values above this threshold couple with a continuous increase in outcome class IV, apparently driven by the decrease in outcomes I and II, since the value of outcome III reaches a plateau.

Similarly, OAI values ranging from 0 to ~20/30 weeks, are associated with a drastic reduction to a null plateau of the probability of outcome I, matched by a decrease in probability of outcome II and an increase in the probabilities of outcomes III and IV. OAI values above 50 weeks couple with an increase in in-hospital death, driven by a reduction in outcome III, given the almost null values estimated for outcomes I and II. However, given the skewed distribution of OAI values, few subjects exhibit measures above 30 wks, therefore, it is wise to hold the judgement on outcome behavior in this range and consider the results as a suggestion.

Improved values of the Level of Cognitive Functioning Scale (LCFS) scores above 1 are matched by greater probabilities of outcomes I and II and a reduced probability of outcomes III and IV. These changes exhibit a marked non-linear relationship and are limited to a small interval (between 1 and 3) of the span of LCFS scores. All probabilities remain unchanged for LCFS scores above 3.

Reduced GOSE at rehabilitation admission is associated with an increase in outcomes III and IV and a reduction in outcome II. However, it is notable that changes in GOSE do not capture variations in outcome I. Considered the good results obtained checking the performance of the prognostic model (see S1 Appendix) it is probable that the observed behavior reflects the GOSE/outcome relationship more than a biased estimation.

Fig 1F shows that the presence of PEG/NGI is linked to a greater probability of outcomes III and IV and a lower probability of outcomes I and II.

In Fig 1G, the presence of CAD is associated with a consistent increase in outcome IV, while there is a decrease in outcomes II and III, and outcome I shows an unexpected increase.

### Multivariable analysis of long-term outcome

**Fig 2:**
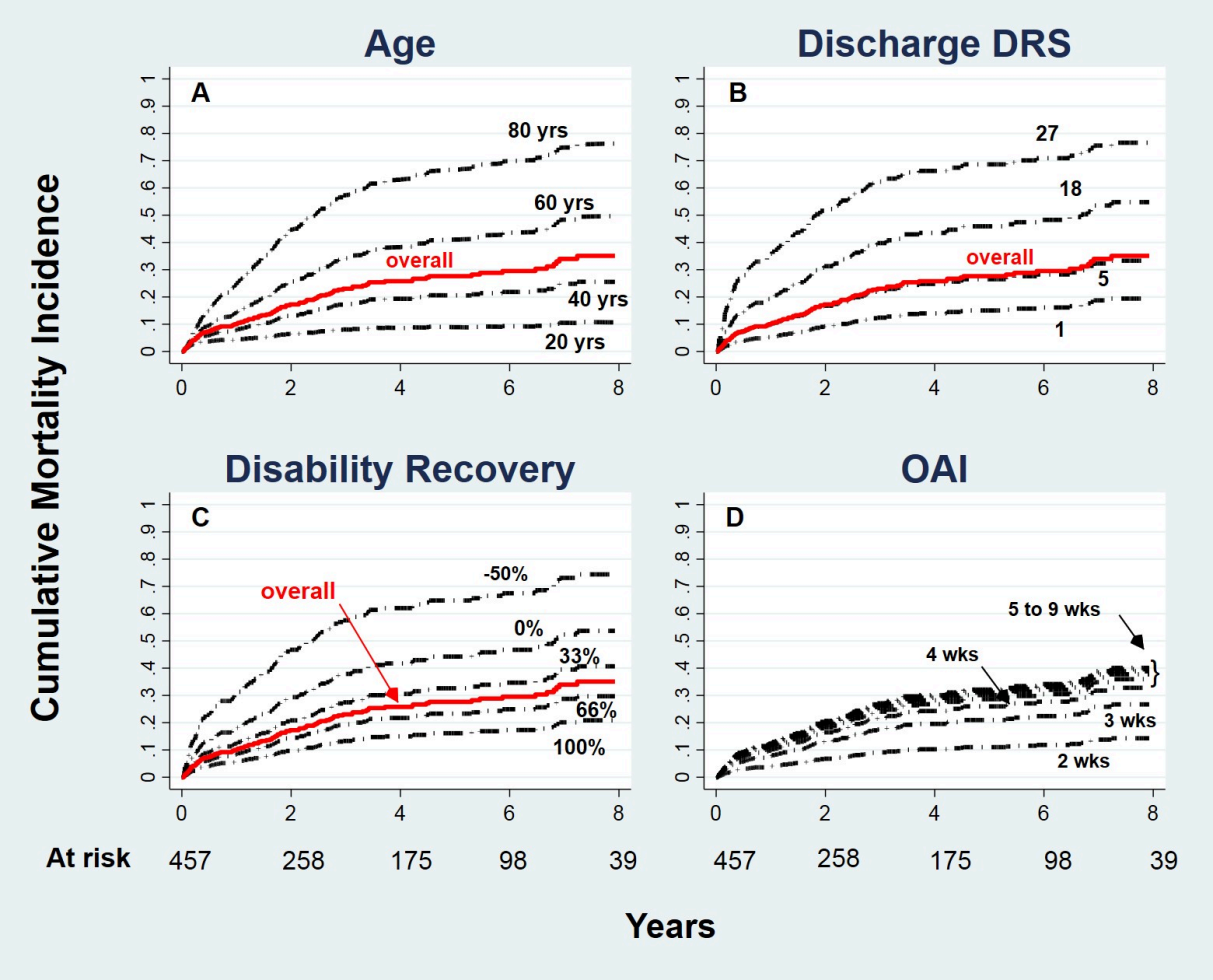
Cox cumulative mortality incidence. (A-D) Population-level adjusted mortality curves (*dashed black lines*). Overall mortality incidence (*red line*). (D) Overall mortality omitted for clarity. DRS = Disability Rating Scale.

Over a mean follow-up of 3.4±2.8 years (range 13 days– 9.8 years) all-cause deaths were 107 (final cumulative mortality rate at 10 years 37.6±3.9%, with 77.2% censoring and number of subjects at risk for each year reported in Fig 2. The results of the final Cox multivariable analysis are shown in Table 4. (Full Cox model results are reported in the S2 Table of the Supporting Information)

**Table 4:** Cox analysis results of long-term outcome.

	Global R^2^ = 0.52—Haller's C = 0.68±0.03						
	Hazard ratio	*p*-value	Percent fraction of global R^2^(%)	Bootstrap inclusion frequency (%)	Linearity stability (%)	Interaction with time	
						Hazard ratio	*p*-value
Age (10 years)	1.37	≤0.0001	42.3	96.2	0.724	1.11	0.03
OAI (1/x2) weeks	0.95	0.04	5	55.2	0.192		NS
Disability recovered	0.29	0.014	23.8	42.1	0.789		NS
Discharge DRS	1.09	≤0.0001	29	84	0.907		NS

OAI: acute onset to admission interval. DRS: Disability Rating Scale. NS: not significant.

Four of the 15 factors tested showed an independent significant association with long-term outcome. The proportionality assumption was rejected only for age and, therefore, an interaction term of age with time was included in the extended final Cox model, which therefore included the OAI interval, the percent of disability recovery during the rehabilitation period, and the residual disability at hospital discharge, as measured with the DRS scale. The goodness-of-fit between the observed and estimated death rates is documented by a non-significant 5-group Gronnesby and Borgan test (*p*-values ranging from 0.5 to 0.78). A global R^2^ of 0.52, along with a Harrell’s C of 0.68, documented the good discriminant ability of the final model, i.e. 52% of the outcome variation (in the log-hazard scale) is explained by the model.

Fig 2 shows the mortality incidence curves, estimated from the final model, relative to various levels of the four factors compared to the observed overall mortality curve. It is notable that each curve is "population-level adjusted", i.e. represents the profile that would be observed if all patients in the study population had the given factor at the chosen level and, thus, together provide a graphical estimate of the impact that each factor exerts on the outcome independent of the other factors. The range of the factor’s level shown was selected within the range observed in the population and the accuracy of each fit depends from the accuracy of the final model (proven to be for the most part adequate: see previous paragraph and S1 Appendix). Naturally, a greater variability is expected to reside on the extreme values but to an extent that does not impair a correct representation of the information embedded in the observed data.

**Fig 3:**
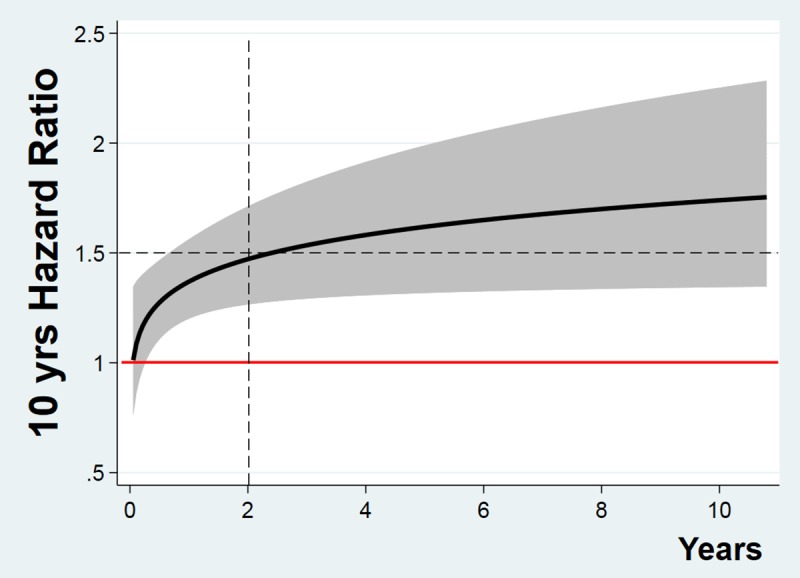
Age hazard ratio. Behavior over time of the age hazard ratio (HR) relative to 10 years increase. Shaded area: 95% confidence interval. See S1 Appendix for details on how to compute HR when time interaction is present.

As expected, increasing age is associated with increasing mortality (Fig 2A). Less obviously, the significant interaction with time implies that, as shown in Fig 3, the effect of age on mortality, as expressed by the specific hazard ratio (HR), increases with time. It is practically null (HR = 1) at hospital discharge, with a rapid increase up to 2 years, followed by a slow plateau at approximately 1.5 HR.

The weight of age on overall mortality is relevant, as suggested by the spread of the age-specific mortality curve in Fig 2A, and as documented by its relevant contribution (42.3%) to the global R^2^ (Table 2).

The second in line, as concerns impact on outcome, is disability status at rehabilitation hospital discharge, which accounts for 29% of the global R^2^ (Fig 2B). Lower values of discharge DRS are associated with favorable mortality profiles and, as expected, lower mortality incidence is associated with greater recovery (during the rehabilitation period) of the disability burden measured at hospital admission (Fig 2C). Interestingly, these two factors, namely the amount of disability at the end of the rehabilitation stage and the percent of disability recovered, account for more than 50% of the overall outcome variation observed.

An increase in the interval between onset of the acute event and admission to the rehabilitation setting is associated with worse outcome (Fig 2D). This association is significantly non-linear (1/x^2^), i.e. an appreciable increase is observed when OAI increases from 2 to 3 weeks, whereas almost no changes occur above 5 weeks.

### Bootstrap sampling (internal validity)

As concern the GOLOGIT analysis of the short-term outcome, the stability of each factor is shown by its frequency of occurrence (bootstrap inclusion frequency; BIF) over the bootstrap replications. BIF values ≥90% imply a solid constancy of the specific factor (Table 3 and S1 Table). Age, OAI, LCFS score, and DRS score show reliable stability. The other significant factors in the final GOLOGIT model exhibit lower values, which reach a value of 65.9% in the case of the presence of CAD.

In the Cox analysis of the long-term outcome the most stable effect on mortality is shown for age (Table 4). Of the bootstrap replicates, 96.2% show this factor as significant, followed by a discharge DRS well above 80%. Regarding the remaining significant factors in the final model, the OAI and the percent of rehabilitation recovery show intermediate stability (55.2% and 42.1%, respectively).

All the factors not included in the final model of both GOLOGIT and Cox analyses show BIF values lower than 30% except for etiology, which, notably, has a value close to 50% for the Cox bootstrap replicates and above 70% for the GOLOGIT replicates (S1 Table and S2 Table).

### Net clinical benefit

**Fig 4:**
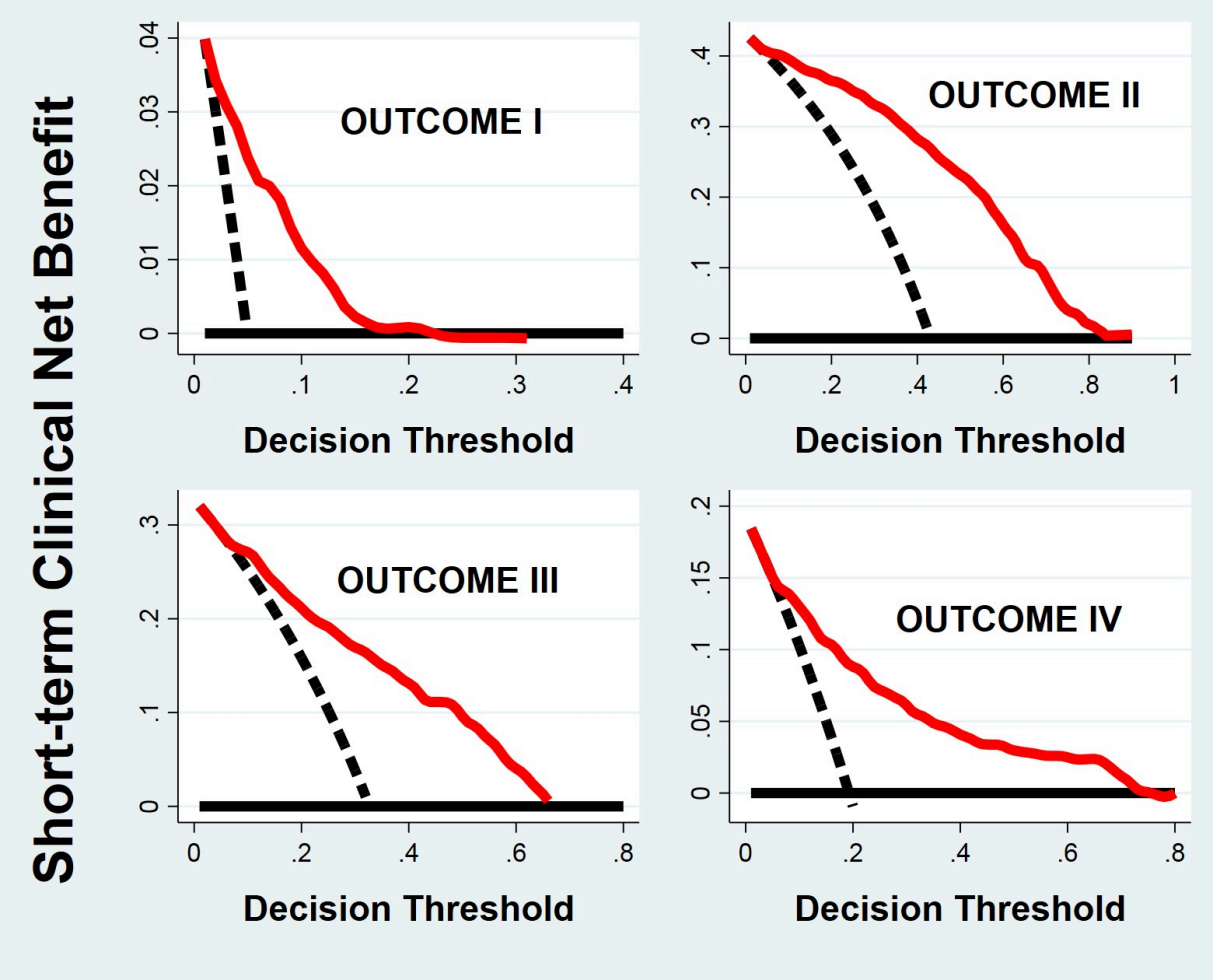
Decision curve analysis of short-term outcome. For each outcome, the net benefit curve of the prognostic model (*red*), assessed using the jack-knife procedure, lies well above the "treat all" (*dashed black*) and "treat none" (*solid black*) curves along a relevant portion of the decision threshold.

The net clinical benefit of using the GOLOGIT prognostic model in the clinical decision-making procedure was computed for each of the four outcomes (Fig 4). The measures, assessed using the jack-knife procedure, result in profiles that are higher than the "treat all" and "treat none" profiles for a relevant interval of the decision threshold, the threshold where the “advantages” of an appropriate clinical action equal the “disadvantages”of a not appropriate clinical action.

**Fig 5:**
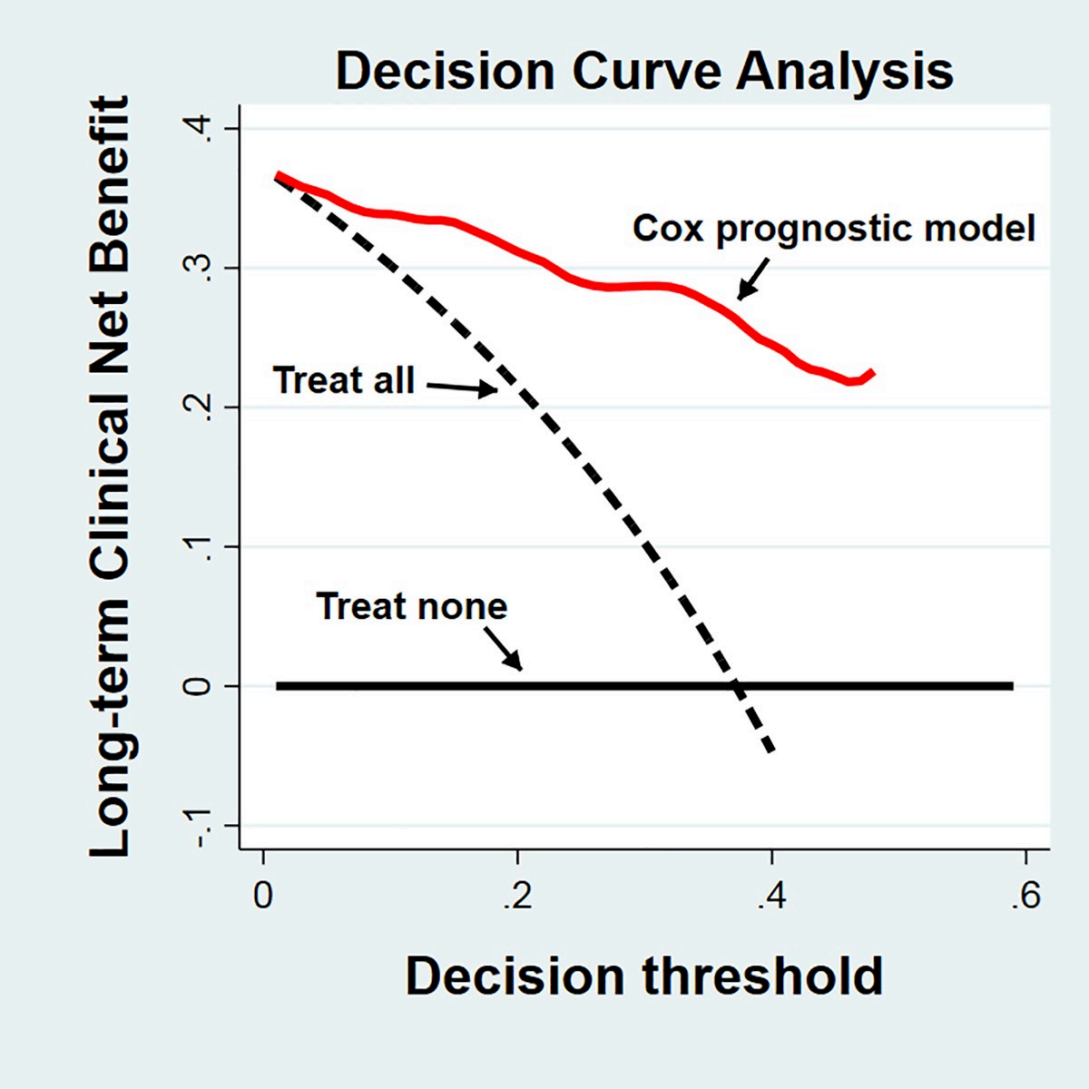
Decision curve analysis of long-term outcome. Net benefit of using the three possible alternatives in the clinical decision-making chain. Use of the prognostic model (*red line*) results in a higher profile than both "treat all" (*dashed black line*) and "treat none" (*continuous black line*) strategies.

Fig 5 shows the net benefit attenable by using the Cox model of long-term outcome as a prognostic tool. Comparably to short-term outcome, the estimated net benefit of making a specific clinical decision according to the Cox model is definitively higher than that evaluated for the two possible alternative choices; applying the given clinical action to all subjects ("treat all") or to none ("treat none") all along the decision threshold range.

## Discussion

This study investigated factors that independently contribute to the short and long-term outcomes in patients with severe ABI using a comprehensive methodology. Our data indicate that both outcomes are mainly associated with age and disability degree (attained and recovered) in two prognostic models that account for a relevant portion of the outcome variability (pR^2^ = 0.32 and R^2^ = 0.52). The role of the factors governing the clinical instability of the acute stage of the disease, as measured by the time spent in the intensive care unit (OAI) [24], is similar on short and long-term outcomes (the partial contribution to the global R^2^ is 5.9% and 5% respectively) and, thanks to the non-linearity of its effect, intervals greater than 5/6 months (short-term outcome, Fig 1C) and 1 month (long-term outcome, Fig 2D) share a flat effect, supporting the existence of a "ceiling effect" for this risk factor in both prognostic models.

The comprehensive analytical method adopted let consider the results from a wider perspective. Considering, together, the statistical significance, the contribution to global R^2^, and the stability (BIF) of each investigated risk factor, it is possible to obtain more details about their association with outcome.

A significant factor may show several combinations of stability levels and weights on the outcome allowing a more exhaustive depiction of the factor’s role in the model.

As example, in the short-term prognostic model, the rehabilitation admission DRS and LCFS (Table 3) show the highest contribution to global pR^2^ and very high BIF values (>95%) thus setting both factors as the most reliable predictors in the final model with the greatest significant impact on outcome, given that, together, they account for almost 45% of the variability in short-term outcome. As shown in Fig 1D changes in LCFS score correlated with changes in short-term outcome probability only for low-score values, suggesting that the clinically useful portion of this scale is limited to less than one-third of the scale.

Similarly, on long-term outcome, psychophysical condition (disability attained at hospital discharge and gained during hospitalization) plays a pivotal role in the long-term prognosis and, in the early period after hospital discharge, accounts for almost all of the variability in the outcome. In fact, age-associated death increases with time and shows only 2 years later an effect on outcome comparable to that of the psychophysical condition (Fig 3).

The lack of statistical significance in the results of both short and long-term outcome suggests the absence of an independent association between etiology and outcome probability. Therefore, the better outcome attributed to some etiologies, namely trauma (1, 2), should be ascribed to the younger age and lesser extent of disability at admission, as documented in our population by the statistically significant lower age and DRS in subjects with traumatic etiology (age 55.1±15.6 vs 43.6±18.8 years, *p*≤0.001; DRS, 20.0±5.2 vs 18.4±6.0, *p* = 0.003). However, the high frequency of a significant association observed in the bootstrap replications (BIF = 71.6% and BIF = 49.5% for short and long-term models respectively (see S1 Table and S2 Table of Supporting Information) may suggest that the traumatic etiology could make an intrinsic contribution to outcome probability independent of the younger age and lower disability that, usually, characterize patients with head trauma. It is likely that a greater power (sample size) would be required to obtain a significant association, considering the multilevel category (we report six different etiologies) and an independent effect that (if present) is relatively small compared to its variability.

It is notable that none of the registered concomitant diseases was found to be statistically significant in the long-term final model and only CAD shows a significant but negligible contribution (partial pR^2^ = 1.3%, Table 3) to short-term association. This observation is reinforced by the low frequency of statistical significance in the associated bootstrapped replications. This unexpected result may be due to the natural selection bias that occurred in the acute stage, i.e. the presence of comorbidities *is* a risk factor and patients surviving to the acute phase come to the rehabilitation hospital after a "selection" that leaves subjects less susceptible to the risk factor itself. This hypothesis is supported by a report documenting that comorbidity is one of the most important determinants of in-hospital mortality for patients with traumatic brain injury, and a significant two-fold increase in the hazard of in-hospital mortality is associated with a Charlson Comorbidity Index score of 1–2 [25].

In order to gain insight into the clinical performance of the final prediction models using a metric that takes into accounts the "cost" as well as the "benefits" of acting according to a given prognostic model, we used DCA to determine the "net benefit" obtained. As shown in Figs 4 and 5, the "net benefit" curves of the four short-term outcomes and of the long-term outcome, estimated using the respective prognostic models, were compared to the curves of the other possible action strategies; "treat all" and "treat none". In all instances, the "net benefits" of using the prognostic models were higher than those of the two alternatives along a broad and meaningful portion of the "decision threshold". Thus, it can be concluded that both final prognostic models have favorable and clinically useful characteristics even when taking into account risk/benefit ratios.

In conclusion, examining our cohort data with integrated analytical tools aimed to estimate not only the statistical significance, but also the weight and the “stability” of prognostic factors as well as the ‘net’ clinical benefit, let us to give a more comprehensive view of the prognostic connections in patients affected by ABI. The independent relevance of the disability condition in the short and long-term prognosis is compared with the weight and “stability” of the other factors considered, and gives back a prognostic framework more accurate and detailed.

### Limitations

Since this is a single-center study, the observations may reflect regional- and environment-related population characteristics. Moreover, our data refer to patients after the acute phase and, thus, the results may apply only to subjects in the same stage.

However, the center covers the 30% of the regional hospital facilities dedicated to the rehabilitation of ABI patients and, therefore, accounts for a non-trivial fraction of the incident pathology in the region (Center- South of Italy) and may be considered representative of the disease.

"Internal validation analysis" provided information on the stability of the results of the current study; however, external validation using population data gathered from other institutions is needed to warrant a valid external inference of these results.

## Supporting information

S1 Checklist STROBE checklist of items that should be included in reports of cohort studies. (DOCX)

S1 Appendix Model-building strategy. (DOCX)

S1 Table Full GOLOGIT results. (XLSX)

S2 Table Full cox results. (XLSX)

S3 Table Dataset short-term. (XLSX)

S4 Table Dataset long-term. (XLSX)

S1 Fig Short-term outcomes ROC curves. Jack-knife corrected receiver operating characteristics curves (ROC) relative to the four outcomes of the final GOLOGIT model. OUT I–IV: outcomes I–IV, AUC: area under the ROC curve.
(TIF)

S2 Fig GOLOGIT calibration plot. Jack-knife corrected calibration plot of observed probabilities vs predicted probabilities computed using jack-knife re-sampling procedure. *Hollow circle* diameters are proportional to group’s size. *Grey shaded area* outlines the frequency of the observed probabilities.
(TIF)

## References

[1] Colantonio A, Gerber G, Bayley M, Deber R, Yin J, Kim H. Differential profiles for patients with traumatic and non-traumatic brain injury. *J Rehabil Med*. 2011 3;43(4):311–5. 10.2340/16501977-0783 21347507

[2] Smania N, Avesani R, Roncari L, Ianes P, Girardi P, Varalta V et al Factors predicting functional and cognitive recovery following severe traumatic, anoxic, and cerebrovascular brain damage. *J Head Trauma Rehabil*. 2013 Mar-Apr;28(2):131–40. 10.1097/HTR.0b013e31823c0127 22333677

[3] Avesani R, Roncari L, Khansefid M, Formisano R, Boldrini P, Zampolini M et al The Italian National Registry of severe acquired brain injury: epidemiological, clinical and functional data of 1469 patients. *Eur J Phys Rehabil Med*. 2013 10;49(5):611–8. 23558700

[4] Shaun Gray D, Burnham RS. Preliminary outcome analysis of a long-term rehabilitation program for severe acquired brain injury. *Arch Phys Med Rehabil*. 2000;81:1447–56. 10.1053/apmr.2000.16343 11083347

[5] Menon DK, Zahed C. Prediction of outcome in severe traumatic brain injury. *Curr Opin Crit Care*. 2009 10;15(5):437–41. 10.1097/MCC.0b013e3283307a26 19713837

[6] Silverberg ND, Gardner AJ, Brubacher JR, Panenka WJ, Li JJ, Iverson GL. Systematic review of multivariable prognostic models for mild traumatic brain injury. *J Neurotrauma*. 2015 4 15;32(8):517–26. 10.1089/neu.2014.3600 25222514

[7] Gao J, Zheng Z. Development of prognostic models for patients with traumatic brain injury: a systematic review. Int J Clin Exp Med 2015 11 15;8(11):19881–5. 26884899 PMC4723744

[8] Maas AI, Murray GD, Roozenbeek B, Lingsma HF, Butcher I, McHugh GS et al; International Mission on Prognosis Analysis of Clinical Trials in Traumatic Brain Injury (IMPACT) Study Group. Advancing care for traumatic brain injury: findings from the IMPACT studies and perspectives on future research. Lancet Neurol. 2013 12;12(12):1200–10. 10.1016/S1474-4422(13)70234-5 24139680 PMC3895622

[9] Cullen NK, Park YG, Bayley MT. Functional recovery following traumatic vs non-traumatic brain injury: a case-controlled study. *Brain Inj*. 2008 12;22(13–14):1013–20. 10.1080/02699050802530581 19117180

[10] Stevens RD, Sutter R. Prognosis in severe brain injury. *Crit Care Med*. 2013 4;41(4):1104–23. 10.1097/CCM.0b013e318287ee79 23528755

[11] Perel P, Edwards P, Wentz R, Roberts I. Systematic review of prognostic models in traumatic brain injury. *BMC Med Inform Decis Mak*. 2006 11 14;6:38 10.1186/1472-6947-6-38 17105661 PMC1657003

[12] Lanzillo B, Matarazzo G, Calabrese C, Vitale DF. Normalization of functional independence measure variation improves assessment of stroke rehabilitation outcome. *Eur J Phys Rehabil Med*. 2015 10; 51(5):587–96. 25573600

[13] Williams R. Generalized ordered logit/partial proportional odds models for ordinal dependent variables. *Stata J*. 2006;6(1):58–82.

[14] Nieto FJ, Coresh J. Adjusting survival curves for confounders: a review and a new method. Am J Epidemiol 1996;143:1059–68. 10.1093/oxfordjournals.aje.a008670 8629613

[15] Royston P, Lambert PC. Flexible parametric survival analysis using Stata:beyond the Cox model. College Station, Texas: Stata Press; 2011 pp 275–282.

[16] Rengo G, Pagano G, Filardi PP, Femminella GD, Parisi V, Cannavo A et al Prognostic Value of Lymphocyte G Protein-Coupled Receptor Kinase-2 Protein Levels in Patients With Heart Failure. Circ Res. 2016 4 1;118(7):1116–24. 10.1161/CIRCRESAHA.115.308207 26884616 PMC4818176

[17] Royston P, Ambler G, Sauerbrei W. The use of fractional polynomials to model continuous risk variables in epidemiology. Int J Epidemiol. 1999;28:964–974. 10.1093/ije/28.5.964 10597998

[18] Shorrocks AF. Decomposition procedures for distributional analysis: a unified framework based on the Shapley value. *J Econ Inequal*. 2013;11:99–126.

[19] Royston P, Sauerbrei W. Multivariate model building. A pragmatic approach to regression analysis based on fractional polynomials for modeling continuous variables. Chichester, UK: Wiley; 2008pp 183–199.

[20] Sauerbrei W, Royston P, Look M. A New Proposal for multivariable modelling of time-varying effects in survival data based on fractional polynomial time-transformation. Biom J. 2007 6;49(3):453–73. 10.1002/bimj.200610328 17623349

[21] Vickers AJ. Decision analysis for the evaluation of diagnostic tests, prediction models and molecular markers. *Am Stat*. 2008;62:314–320. 10.1198/000313008X370302 19132141 PMC2614687

[22] Vickers AJ, Cronin AM, Elkin EB, Gonen M. Extensions to decision curve analysis, a novel method for evaluating diagnostic tests, prediction models and molecular markers. BMC Med Inform Decis Mak. 2008 11 26;8:53 10.1186/1472-6947-8-53 19036144 PMC2611975

[23] von Elm E, Altman DG, Egger M, Pocock SJ, Gøtzsche PC, Vandenbroucke JP; STROBE Initiative. The Strengthening the Reporting of Observational Studies in Epidemiology (STROBE) statement: guidelines for reporting observational studies. *Lancet*. 2007 10 20;370(9596):1453–7. 10.1016/S0140-6736(07)61602-X 18064739

[24] Formisano R, Azicnuda E, Sefid MK, Zampolini M, Scarponi F, Avesani R. Early rehabilitation: benefits in patients with severe acquired brain injury. *Neurol Sci*. 2017 1;38(1):181–184. 10.1007/s10072-016-2724-5 27696274

[25] Fu TS, Jing R, McFaull SR, Cusimano MD. Recent trends in hospitalization and in-hospital mortality associated with traumatic brain injury in Canada: a nationwide, population-based study. *J Trauma Acute Care Surg*. 2015 9;79(3):449–54. 26535433 10.1097/ta.0000000000000733

