# Supplementary material for: Prognosis of severe acquired brain injury: Short and long-term outcome determinants and their potential clinical relevance after rehabilitation. A comprehensive approach to analyze cohort studies
Source: PLoS One. 2019 Sep 26;14(9):e0216507. doi: 10.1371/journal.pone.0216507 (PMC6762165; doi:10.1371/journal.pone.0216507)
Supplement: S1 Checklist — (DOCX) [file pone.0216507.s001.docx]

**STROBE Statement**—checklist of items that should be included in reports of observational studies

|  | **Item No** | **Recommendation** |
| --- | --- | --- |
|  |  |  |
| **Title and abstract** | 1 | *(a)* Indicate the study’s design with a commonly used term in the title or the abstract |
|  |  | **In the title is indicated the study design** |
|  |  | *(b)* Provide in the abstract an informative and balanced summary of what was done and what was found |
|  |  | **The abstract contains the following sections: background, methods & findings, and conclusions** |
| **Introduction** |  |  |
| Background/rationale | 2 | Explain the scientific background and rationale for the investigation being reported |
|  |  | **See Introduction, paragraphs 1–4** |
| Objectives | 3 | State specific objectives, including any prespecified hypotheses |
|  |  | **See Introduction, paragraphs 5-7** |
| **Methods** |  |  |
| Study design | 4 | Present key elements of study design early in the paper |
|  |  | **Presented in Abstract, “Methods and Findings”; and in Methods, “Study population” sub-section** |
| Setting | 5 | Describe the setting, locations, and relevant dates, including periods of recruitment, exposure, follow-up, and data collection |
|  |  | **Described in Methods, “Study population” sub-section** |
| Participants | 6 | *(a) Cohort study*—Give the eligibility criteria, and the sources and methods of selection of participants. Describe methods of follow-up |
|  |  | **Done in Methods, “Study population” sub-section** |
|  |  | *Case-control study*—Give the eligibility criteria, and the sources and methods of case ascertainment and control selection. Give the rationale for the choice of cases and controls |
|  |  | **N/A** |
|  |  | *Cross-sectional study*—Give the eligibility criteria, and the sources and methods of selection of participants |
|  |  | **N/A** |
|  |  | ***(b)*** *Cohort study*—For matched studies, give matching criteria and number of exposed and unexposed |
|  |  | **N/A** |
|  |  | *Case-control study*—For matched studies, give matching criteria and the number of controls per case |
|  |  | **N/A** |
| Variables | 7 | Clearly define all outcomes, exposures, predictors, potential confounders, and effect modifiers. Give diagnostic criteria, if applicable |
|  |  | **See Methods, “Study population” and “ Statistical Analysis” sub-sections; see S2 Appendix Model building strategy.** |
| Data sources/ measurement | 8* | For each variable of interest, give sources of data and details of methods of assessment (measurement). Describe comparability of assessment methods if there is more than one group |
|  |  | **See Methods, “Study population” and S2 Appendix Model building strategy.** |
| Bias | 9 | Describe any efforts to address potential sources of bias |
|  |  | **See Discussion “Limitations” sub-section; see S2 Appendix Model Building Strategy “Internal validity” sub-section** |
| Study size | 10 | Explain how the study size was arrived at |
|  |  | **Described in Methods, “Study population” sub-section** |
| Quantitative variables | 11 | Explain how quantitative variables were handled in the analyses. If applicable, describe which groupings were chosen and why |
|  |  | **See Methods, “Study population” and “ Statistical Analysis” sub-sections** |
| Statistical methods | 12 | *(a)* Describe all statistical methods, including those used to control for confounding |
|  |  | **See Methods “ Statistical Analysis” sub-section and S2 Appendix Model Building Strategy** |
|  |  | ***(b)*** Describe any methods used to examine subgroups and interactions |
|  |  | **See Methods “ Statistical Analysis” sub-section and S2 Appendix Model Building Strategy** |
|  |  | ***(c)*** Explain how missing data were addressed |
|  |  | **No missing data** |
|  |  | *(d) Cohort study*—If applicable, explain how loss to follow-up was addressed |
|  |  | **One patient was lost to long-term follow-up and was not considered in the analysis. See Methods “Study Population” sub-section.** |
|  |  | *Case-control study*—If applicable, explain how matching of cases and controls was addressed |
|  |  | **N/A** |
|  |  | *Cross-sectional study*—If applicable, describe analytical methods taking account of sampling strategy |
|  |  | **N/A** |
|  |  | *(e)* Describe any sensitivity analyses |
|  |  | **See S2 Appendix Model Building Strategy “Sensitivity Analysis”** |
| **Results** |  |  |
| Participants | 13* | (a) Report numbers of individuals at each stage of study—eg numbers potentially eligible, examined for eligibility, confirmed eligible, included in the study, completing follow-up, and analysed |
|  |  | **See number at risk of Fig 2** |
|  |  | (b) Give reasons for non-participation at each stage |
|  |  | **N/A** |
|  |  | (c)  Consider use of a flow diagram |
|  |  | **N/A** |
| Descriptive data | 14* | (a)  Give characteristics of study participants (eg demographic, clinical, social) and information on exposures and potential confounders |
|  |  | **See Tabe 1 and 2** |
|  |  | (b) Indicate number of participants with missing data for each variable of interest |
|  |  | **No missing data** |
|  |  | (c)  *Cohort study*—Summarise follow-up time (eg, average and total amount) |
|  |  | **See Results “Multivariable analysis of long-term outcome” sub-section.** |
| Outcome data | 15* | *Cohort study*—Report numbers of outcome events or summary measures over time **See Tabe 1 and 2; Fig1 and 2** |
|  |  | *Case-control study—*Report numbers in each exposure category, or summary measures of exposure |
|  |  | **See Tabe 1 and 2** |
|  |  | *Cross-sectional study—*Report numbers of outcome events or summary measures |
|  |  | **N/A** |
| Main results | 16 | *(a)* Give unadjusted estimates and, if applicable, confounder-adjusted estimates and their precision (eg, 95% confidence interval). Make clear which confounders were adjusted for and why they were included |
|  |  | **See Tabe 1 and 2; Fig1 and 2** |
|  |  | *(b)* Report category boundaries when continuous variables were categorized |
|  |  | **N/A** |
|  |  | ***(c)*** If relevant, consider translating estimates of relative risk into absolute risk for a meaningful time period |
|  |  | **N/A** |
| Other analyses | 17 | Report other analyses done—eg analyses of subgroups and interactions, and sensitivity analyses |
|  |  | **See S2 Appendix Model Building Strategy “Sensitivity Analysis”** |
| **Discussion** |  |  |
| Key results | 18 | Summarise key results with reference to study objectives |
|  |  | **Done in Discussion paragraph 1** |
| Limitations | 19 | Discuss limitations of the study, taking into account sources of potential bias or imprecision. Discuss both direction and magnitude of any potential bias |
|  |  | **Done in Discussion “Limitations” sub-section** |
| Interpretation | 20 | Give a cautious overall interpretation of results considering objectives, limitations, multiplicity of analyses, results from similar studies, and other relevant evidence |
|  |  | **Done in Discussion last paragraph** |
| Generalisability | 21 | Discuss the generalisability (external validity) of the study results |
|  |  | **Done in Discussion “Limitations” sub-section** |
| **Other information** |  |  |
| Funding | 22 | Give the source of funding and the role of the funders for the present study and, if applicable, for the original study on which the present article is based |
|  |  | **N/A** |
