# Supplementary material for: Prognosis of severe acquired brain injury: Short and long-term outcome determinants and their potential clinical relevance after rehabilitation. A comprehensive approach to analyze cohort studies
Source: PLoS One. 2019 Sep 26;14(9):e0216507. doi: 10.1371/journal.pone.0216507 (PMC6762165; doi:10.1371/journal.pone.0216507)
Supplement: S1 Appendix — (DOCX) [file pone.0216507.s002.docx]

**S1 Appendix. Model-building strategy**

In order to model the potential independent effect on the categorical ordered short-term outcome (i.e. the four possible classes of disability at hospital discharge) a generalized ordered logistic model for ordinal dependent variables (GOLOGIT) was adopted, as implemented by the *gologit2* module of STATA (1).

The Cox proportional hazard analysis was used to model long-term outcome.

A multivariable model-building procedure was carried out for both models, following a pragmatic approach proposed by Royston and Sauerbrei (2), summarized as follows.

*Criteria used to choose candidate factors.*

Considering the main aim of the 2 models (to assess the association of patient’s characteristics at admission on outcome at discharge (short-term outcome) and to assess prognostic relevance on long-term outcome of patient’s discharge status) a pool of variables with known or potential capability to influence the outcomes was selected. Parsimonious selection criteria were used to avoid overfitting bias.

The rule of "at least five observed events for each tested variable" (3) was adopted for the GOLOGIT model (events as the classes of disability at hospital discharge). The 19 factors thought to compose the pool of potential predictors are listed in Table 1 in the main article. In order to avoid multicollinearity, a correlation coefficient >0.8 between any two continuous variables prompted concern of multicollinearity. As shown in Table A, none of the correlation coefficients exceeded that threshold. Thus, all 19 factors were used in the multivariable analysis. Their multicollinearity, as measured by the variance inflation factor, was ≤3.3, indicating acceptable levels of variance inflation (4, 5).

| Table A: Matrix correlation of admission continuous variables (r). | | | | | | | |
| --- | --- | --- | --- | --- | --- | --- | --- |
|  | Age | OAI | LCFS score | DRS score | FIM^TM^ motor score | FIM^TM^ cognitive score | GOSE score |
| Age | 1 |  |  |  |  |  |  |
| OAI | –0.004 | 1 |  |  |  |  |  |
| LCFS score | –0.236 | –0.151 | 1 |  |  |  |  |
| DRS score | 0.3124 | 0.1588 | –0.752 | 1 |  |  |  |
| FIM^TM^ motor score | –0.242 | –0.07 | 0.4079 | –0.513 | 1 |  |  |
| FIM^TM^ cognitive score | –0.271 | –0.137 | 0.7176 | –0.694 | 0.5808 | 1 |  |
| GOSE score | –0.282 | –0.146 | 0.6591 | –0.7 | 0.4891 | 0.5699 | 1 |
| OAI: acute onset to admission interval. DRS: Disability Rating Scale. LCFS: Level of Cognitive Functioning Scale. FIM^TM^: Functional Independence Measure. GOSE: Extended Glasgow Outcome Scale. | | | | | | | |

The candidate variables to be tested in the Cox model were selected from the list of the 23 factors reported in Table 2 of the main article. The list includes indexes reflecting the behavior during the rehabilitation stage based on the admission/discharge variation of the five disability measures used. These indexes may be regarded as the gain observed during the rehabilitation hospital stay, and are measured as a percent of the theoretical maximal gain that could be achieved at hospital admission for each disability score. We have previously observed (6) that this normalization procedure of the admission/discharge variation avoids the "ceiling effect" and combines with improved predictive properties of the measure. Multicollinearity was checked by inspecting the correlation matrix of the continuous factors at hospital discharge. Considering that the "percent rescue gained during hospitalization" is a measure derived from each of the five disability scales and that, accordingly, multicollinearity is inherent in the variable construction, these derived factors were not considered for multicollinearity check in the first instance and were excluded from the correlation matrix (Table B).

| Table B. Correlation matrix of discharge continuous variables (r) | | | | | | | | |
| --- | --- | --- | --- | --- | --- | --- | --- | --- |
|  | Age | LOS | OAI | DRS score | LCFS score | FIM^TM^ motor score | FIM^TM^ cognitive score | GOSE score |
| Age | 1 |  |  |  |  |  |  |  |
| LOS | 0.1288 | 1 |  |  |  |  |  |  |
| OAI | –0.018 | 0.2226 | 1 |  |  |  |  |  |
| DRS score | 0.3554 | 0.5379 | 0.2627 | 1 |  |  |  |  |
| LCFS score | –0.2249 | –0.5321 | –0.2468 | –0.8579 | 1 |  |  |  |
| FIM^TM^ mot. | –0.3952 | –0.5239 | –0.2539 | –0.8571 | 0.7031 | 1 |  |  |
| FIM^TM^ cogn. | –0.3315 | –0.5026 | –0.2395 | –0.8535 | 0.8356 | 0.8023 | 1 |  |
| GOSE score | –0.3916 | –0.4849 | –0.2289 | –0.8487 | 0.7243 | 0.8432 | 0.7625 | 1 |
|  |  |  |  |  |  |  |  |  |
| LOS: length of hospital stay, OAI: acute onset to admission interval, DRS: Disability Rating Scale, LCFS: Level of Cognitive Functioning Scale, FIM^TM^: Functional Independence Measure,: mot. : motor score, cogn. : cognitive score, GOSE: Extended Glasgow Outcome Scale. | | | | | | | | |

Almost all pairs of correlations between disability measures, namely the Disability Rating Scale (DRS), the Level of Cognitive Functioning Scale (LCFS), the Extended Glasgow Outcome Scale (GOSE), and both the cognitive and motor domains of the Functional Independence Measure (FIM^TM^) score, show coefficients >0.8. Moreover, the variance inflation factor (VIF) measured on the full set of candidate variables evidenced VIF values between 4.5 and 8.5 for the group of disability measures, indicating the presence of multicollinearity (4,5). Therefore, in order to avoid the estimation bias introduced by high collinearity, we sought to test only the DRS out of the five scale indices. The criteria used to make this selection were:

- the DRS score has a wider range than that of LCFS and GOSE scores, thus potentially allowing a more effective "capture" of the clinical status of patients.
- the DRS score is highly correlated with both cognitive and motor FIM^TM^ scores (r=0.85 and 0.86, respectively; Table B) and, thus, condenses a relevant portion of the information carried out by the two scores.

VIF analysis of the full set of candidate factors including only the DRS score showed a VIF maximum value equal to 1.9, documenting levels of multicollinearity well below the critical threshold (4,5).

Consistent with these multicollinearity considerations, in order to incorporate a “gain” measure in the pool of candidate factors for long-term outcome modelling, we included only the “DRS gain” in the list of variables to be tested in the Cox model. As expected, the maximum VIF observed increased to 5.1; however, it was considered acceptable, given that the VIF threshold of "concern" for multicollinearity is not exactly fixed, and ranges between the most restrictive five to the more relaxed ten (5).

Therefore, 15 factors, marked with an asterisk in Table 1 of the main paper, established the pool of variables tested in the Cox analysis.

*Multivariable analysis.*

As stated, the GOLOGIT and the Cox procedures were used to model short and long-term outcome respectively.

1. The first aim of the GOLOGIT analysis is the selection of the "important" predictors of short-term outcome from the initial pool, using a stepwise selection method. The second aim is the clarification of the functional form (linearity or non-linearity) of the predictor-outcome relationships. This second aim has two aspects. The first is whether the *parallel/non-parallel lines* assumption holds for all variables and can be resolved with the flexibility of the *partial proportional odds* option of the gologit2 module (i.e. this is equivalent to testing whether the coefficients of a given factor are equal or not over the *M*-1 (see next paragraph) logistic equations estimated by GOLOGIT). The second is whether the model is best fitted by assuming a non-linear relationship for continuous variables, such as the square root, the cubic, the logarithm or other non-linear transformation. To this end, the *partial proportional odds* option of the gologit2 module was combined with the multivariable fractional polynomial (MFP) modelling algorithm developed by Sauerbrei and Royston (7), resulting in a final model that combines the exclusion of "unimportant" variables with the selection of a "reasonable" dose-response function for continuous variables. The *p*-value was set to ≤0.05 for variable selection and for testing between variable transformations.

The GOLOGIT model is equivalent to a series of binary logistic regressions in which the M disability classes of the dependent variable (outcome) are combined (e.g. if M=4, as in our study, then there are J=M–1=3 binary logistic series, namely, for J=1 category I is contrasted with categories II, III and IV merged; for J=2 the contrast is between categories I and II vs III and IV; and for J=3, it is categories I, II and III vs category IV). Thus, the output of a GOLOGIT analysis for a four-class outcome comprises the coefficients of these three sets of binary logistic regressions. Given the three series of significant coefficient in the final model, the probabilities (P) computation of each of the (M) four possible outcomes relative to a given set of predictors (X) is derived as follows:

$P(M=I/X)=1-G(X\beta\_1)$

$P(M=II/X)=G(X\beta\_1 )-G(X\beta\_2)$

$P(M=III/X)=G(X\beta\_2 )-G(X\beta\_3)$

$P(M=IV/X)=G(X\beta_{3} )$

where G(β) refers to the three binary logistic regression sets. The full output of the final GOLOGIT model is shown in S1 Table. Parameters of the non-significant factors are the values that would be observed when added next to the significant variables.

Two elements require further clarification:

The partial proportional odds option allows non-linear modelling by testing whether a better fit of the model can be achieved by assigning different values to the coefficients of a given significant factor across the three sets of logistic regressions. If so, it implies that the effect of that factor is not constant over the three sets and, hence, gives rise to a non-linear trend of the derived outcome probabilities. Fig 1A and Fig 1C of the main article are representative, the first, of equal value coefficients (i.e. coefficients for age do not change) and, the second, of changing values (i.e. coefficients for the “interval between onset of the acute event and admission to the rehabilitation setting” are different in the three logistic regression sets). This non-linearity modelling adds up to a test for the inclusion of non-linear transformations of the continuous variables performed by the MFP algorithm. Fig 1D shows a strong non-linear trend of the admission LCFS significantly modelled by both, different coefficient values over the three binary logistic regression sets and by inverse square (1/x2) transformation of this factor.

The weight of each significant factor in the final model was evaluated by the contribution to the global goodness-of-fit, as measured by McFadden’s global pseudo R^2^ (pR^2^). Its partition over the significant predictors was accomplished by the Shapley-Owen decomposition algorithm (8). Interpretation of the values of the pR^2^ considers the fact that its values tend to be lower than those observed for the classical R^2^ calculated by linear regression with ordinary least square (OLS) (i.e. pR^2^ range 0.2–0.4 corresponds to range 0.42–0.78 of OLS R^2^ and indicates excellent model fit) (9).

The discrimination ability of the final prognostic model was estimated by the area under the curve (AUC) of the receiver operating characteristics (ROC) analysis. The ROC curves and the relative AUC of the four predicted outcomes were computed using the jack-knife re-sampling procedure (sample one out) to avoid the “self-reference” bias of the standard analysis and are shown in S1 Figure.

Calibration of the final model, i.e. the agreement between the observed probabilities and the jack-knife predicted probabilities, was assessed with the Hosmer-Lemeshow goodness-of-fit test on eight groups according to the predicted probabilities values (one more than the number of independent variables in the final model (10)). The calibration plot is shown in S2 Figure.

Understanding the GOLOGIT analysis from the numerical results of the analysis is quite demanding (see S1 Table). Therefore, the results of the present study are reported as graph of the probability of each outcome relative to a clinically meaningful interval of values for a significant continuous factor, or as a bar plot of each outcome probability relative to every value for a significant categorical factor. In each plot, the probabilities are “population adjusted” (11) for the confounding effect of the other significant factors of the final model and may be interpreted as the probabilities that would be observed if all subjects in the study population had the given value of the factor under consideration. This results in a visual evaluation of the impact on each outcome of the variation in the given factor independent of the others factors. In particular, the steps to compute the 4 classes outcome behavior relative to a given factor span (e.g. age 20-80) are as follows.

- Estimate the 4 outcome class probabilities for each subject in the dataset using the coefficients of the final GOLOGIT model with the observed factor’s values of each given subject excluding the one for age that is set to 20 for all subjects.
- Compute the mean of the 4 outcome class probabilities and label them to age 20.
- Repeat the procedure for every year in the 20-80 interval.

This gives the results plotted in the manuscript Fig 1A.

It has to be noticed that if the computations are made using the observed factors values of each subject (i.e. without setting a fixed age value) the resulting 4 outcome class averages actually would be the 4 outcome class frequencies observed in the population.

1. The proportional Cox analysis too was carried out with the MFP algorithm in order to assess a “final model” of factors significantly associated to the long-term outcome (mortality) with the most suitable functional form (linearity/non-linearity).

The weight of each significant factor in the final model was evaluated by both the hazard ratio and by the contribution to the global explained variance (R^2^). The hazard ratio (HR) of continuous factors was computed for meaningful variations in the variable (e.g. 10-year period for age) and the partition of the global R^2^ was accomplished by the Shapley-Owen decomposition algorithm (8).

The proportionality assumption of the Cox analysis was tested with a modified version of the MFP procedure (the MFPT), exploring the interaction time/variable(s) of the final model (12). As reported in the main paper, only age showed a significant interaction with time, and prompted the inclusion of the interaction term in the final model as the product of age by the natural logarithm of time (extended Cox model). Computation of the HR relative to 10 years of age increase, taking into account the presence of time interaction, was made using the following formula:

$$e^{\beta_{1}+\beta_{2}\times ln(t)}$$

Where β_1_ is the coefficient of Age (ln(1.37)=0.315) and β_2_ is the coefficient of the interaction term (Age*ln(time)) and evaluates to ln(1.11)=0.104, with time *t* expressed in years.

Details of the adopted procedure are reported by P Royston & PC Lambert (13).

The full output of the final Cox model is shown in S4 Table. Parameters of the non-significant factors are the values that would be observed when added next to the significant variables.

We assessed calibration and the discrimination ability of the final model. The calibration, i.e. the concordance between the observed and model estimated mortality, was assessed by the Gronnesby and Borgan test over five risk groups. Non-significant tests indicate good calibration (14). The discrimination ability was tested by both the Harrell’s C (as an extension of the binary logistic C statistic corrected for the censoring bias (15)) and by the explained variance in the natural scale of the Cox model (R^2^) as proposed by Royston and Sauerbrei (16).

In order to enable interpretation of the role and weight of each factor in the overall outcome, mortality curves adjusted according to the final Cox model were constructed. The normalization procedure is based on the "directly adjusting method" (11), whereby each adjusted curve is constructed by applying the coefficients of the final Cox model to the factor values observed in each patient of the population, then averaging the mortality curves so obtained (“population adjusting”). Thus, utilization of the factor values observed in each subject results in an (averaged) mortality curve that mimics the derived mortality from the classical overall Kaplan–Meier curve to such an extent that, in our data, the numerical difference between the two curves is ≤ the 5th significant digit. Making adjustment after substitution of specific values for a given factor (e.g. assign to all patients an age of 40 years), results in a curve that estimates the shape that would be observed if all patients had the chosen value (e.g. as if all patients were 40 years old). Repeating this procedure for values of interest for specific factors improves the interpretation of the results of the Cox analysis (see Fig. 2 in the main article).

*Internal validity*

The internal validity of both models was computed by assessing the stability of each model characteristics using non-parametric bootstrap sampling (chapter 2.2, p. 24 (2)). Given the coefficients estimated with the GOLOGIT model, using the described model-building procedure, the stability of each factor tested in the model was measured by the frequency that this factor was selected as "significant" in a large series (1000) of bootstrap replications of the dataset by applying the same procedure. Each bootstrapped dataset may be considered as a random replicate of the original dataset; thus, the bootstrap inclusion frequency (BIF) of a given factor in the final model quantifies the confidence we can place on its association with the outcome (stability), considering the expected random variability of the data. Similarly, the stability of the functional form of a continuous factor/outcome relationship is measured by the frequency of a significant linear vs non-linear occurrence on the bootstrap subsample.

The same bootstrap procedure with 1000 replications was applied to The Cox analysis.

S1 Table reports the BIF for each variable tested and the linear functional form frequency (LF) of the significant continuous factors for the GOLOGIT analysis. S2 Table shows the corresponding results for the Cox analysis.

*Clinical Utility (Decision Curve Analysis)*

Sensitivity, Specificity and other statistical measures such as the area under the ROC curve do not give a direct answer on how prognostic tools perform when translated in the clinical practice (17). The extent of clinical impact that would results from their application in the routine management of patients depends also from the type, “efficiency” and possible side effects of the clinical action driven by the knowledge of each patient prognosis. Therefore, ad hoc studies are required to measure the advantage obtained by using a given prognostic tool by assessing “cost” and “benefits” peculiar to the given “action” considered, the action being a drug, a clinical procedure, or any practice aimed to improve the prognosis of the patient. Given this scenarios variety, the specific information required are not available in the data used to derive the prognostic model, moreover, clinical decision have, often, to be taken taking into account the different acceptance of the trade-off between Advantages and Disadvantages (perceived A/D ratio) of a clinical action by the single patient (or physician). A.J. Vickers (18-20) proposed a procedure, the DCA analysis, able to give an idea of how the prognostic model may perform when applied in the clinical environment. The method rests on subtracting from the “benefit” of a “nonspecific” clinical action its “cost” weighted by the measure of all possible individual trade-off preferences using a metric derived only by the data available for model development. The “clinical action” does not needs to be specified and the metrics are derived from the fact that is taken or not. The formula follows.

$$Net Benefit=Benefit-Cost\times odds(t)$$

The logic of the procedure is; given a clinical action of any kind aimed to patients with a given status of the disease or with a given event like the death, i.e. “cases”, given a prognostic tool able to identify “cases” by estimating the probability of being a “case” and, thus, able to constructing a rule for case/no-case targeting by selecting a threshold with a given accuracy, the “benefit “ is measured by the frequency of “appropriate” clinical action, that is the frequency in the population of the patients that being “cases” are identified by the prognostic tool as “cases” (true positive) and, thus, are “treated” with the clinical action. The “cost” is measured by the frequency in the population of “non-appropriate” clinical action, i.e. the frequency of subjects that being targeted incorrectly as “cases” by the prognostic tool (since they are “not cases”, i.e. false positive) are exposed to the clinical action.

The subtraction of the “cost” from “benefit”, so far, imply that cost and benefit have the same weight or, in other words, that one appropriate clinical action is equivalent to one non-appropriate clinical action (i.e. an Advantage/Disadvantage ratio of 1 to 1). Since this is rarely the case the “cost” term has to be weighted by some estimate of the Advantage/Disadvantage ratio.

In the DCA analysis the “cost” is multiplied by the odds of the threshold used to identify “cases” since the threshold itself imply Advantage/Disadvantage ratio estimate. E.g. selecting a threshold of 0.20 means that at the probability of 20% the advantages of being treated as a “case”, in the event of being indeed a case, equals the disadvantages of being treated as a “case” in the event of not being a case. This means that 4 not-appropriately treated “not case” (“cost”) worth 1 appropriately treated “case” (“benefit”) or, equivalently, that one “cost” weight 1/4 of one “benefit”. Therefore, a reasonable estimate of the weight to be assigned to the “cost” is the odds of the threshold “t” (odds of 0.2=0.2/(1-0.2)=1/4). The extension to other decision thresholds is straightforward, e.g. a threshold of 0.5 imply equal weight of “cost” and “benefit” (odds of 0.5=1), while a threshold of 0.8 imply that “cost” weighs 4 times “benefit” (odds of 0.8=0.8/(1-0.8)=4).

To have an exhaustive representation, the DCA analysis computes the net benefit relative to all possible thresholds observed in the population and compares the resulting curve with that obtained applying the clinical action to all subjects irrespective of the prognostic tool (“treat all”) and to that obtained not applying the clinical action to any of the subjects (“treat none”). The net benefit for this last curve, obviously, equal 0.0 for all threshold’s values.

It is worth noting that, at any threshold, the following mathematic equivalences hold (21):

$$Benefit=Sensitivity\times P$$

and

$$Cost=(1-Specificity)\times(1-P)$$

where P is the prevalence of “cases” in the population.

The Author of the DCA procedure makes available at:

https://www.mskcc.org/departments/epidemiology-biostatistics/biostatistics/decision-curve-analysis

the pertinent references, the code to make the analysis in STATA, R and SAS and a tutorial to run it.

Computation of net benefit requires the estimation of outcome probabilities using the final model for each subject in the study population. This implies that we have to account for an "optimistic bias", since the probability of each subject is computed using a model built using the peculiarities of the individual subject. To avoid the indicated bias, the net benefit was computed using the jack-knife procedure, i.e. the outcome probabilities of a given subject were estimated using a model built excluding the subject under assessment.

*Sensitivity Analysis*

The discharge from the rehabilitation hospital represents the threshold delimiting in-hospital death and long-term mortality. It can be argued that deaths occurring soon after hospital discharge may change short and long-term prognostic model characteristics if considered as occurred in-hospital. To ascertain how much sensible the model behavior was to 1-month shift of the hospital discharge, we recomputed both models simulating as in-hospital the deaths occurring within 1 month from discharge.

Seven (3.2%) out 216 deaths occurred in the 1^st^ moth after hospital discharge.

The numerical variation between original model and model with 1-month shift of in-hospital deaths of the statistically significant coefficients were less or equal to the 10% of the original. When Odds Ratio (OR, short-term model) and Hazard Ratio (HR, long-term model) scale were used the variation was less or equal to 5%.

Since judgment based on numerical coefficients (or OR&HR) is quite difficult, we compared the graphs of Fig 1 and Fig 2 of the manuscript with the same graphs obtained using the models with 1-month shift. Visual inspection of enlarged paired plots did not show appreciable differences.

We conclude that the results observed are not sensitive to reassignment of the deaths occurring within 1 month of hospital discharge.

**Appendix References**

1. Williams R. Generalized ordered logit/partial proportional odds models for ordinal dependent variables. Stata J 2006;6(1):58-82.
2. Royston P, Sauerbrei W. Multivariate model building. A pragmatic approach to regression analysis based on fractional polynomials for modeling continuous variables. Chichester, UK: Wiley; 2008.
3. Vittinghoff E, McCulloch CE. Relaxing the rule of ten events per variable in logistic and Cox regression. Am J Epidemiol 2007 Mar 15;165(6):710-8.
4. Kutner MH, Nachtsheim CJ, Neter J, Li W. Applied linear statistical models. 5th edition. New York. NY: McGraw Hill/Irwin; 2005: 409.
5. Suleiman AA. Analysis of multicollinearity in multiple regressions. Int J Adv Technol Eng Sci (www.IJATES.com) 2015;3(1):571-578.
6. Lanzillo B, Matarazzo G, Calabrese C, Vitale DF. Normalization of functional independence measure variation improves assessment of stroke rehabilitation outcome. Eur J Phys Rehabil Med. 2015 Oct;51(5):587-96.
7. Royston P, Ambler G, Sauerbrei W. The use of fractional polynomials to model continuous risk variables in epidemiology. Int J Epidemiol 1999;28:964–974.
8. Shorrocks AF. Decomposition procedures for distributional analysis: a unified framework based on the Shapley value. J Econ Inequal 2013;11:99–126.
9. Domencich T, McFadden DL. Urban travel demand: a behavioral analysis. Amsterdam: North-Holland Publishing Co., 1975. Reprinted 1996. Available from: https://eml.berkeley.edu/~mcfadden/ travel.html
10. Lemeshow S, Hosmer DW Jr. A review of goodness of fit statistics for use in the development of logistic regression models. Am J Epidemiol 1982 Jan;115(1):92-106.
11. Nieto FJ, Coresh J. Adjusting survival curves for confounders: a review and a new method. Am J Epidemiol 1996;143:1059-68
12. Sauerbrei W, Royston P, Look M. A new proposal for multivariable modelling of time-varying effects in survival data based on fractional polynomial time-transformation. Biom J 2007 Jun;49(3):453-73.
13. Royston P, Lambert PC. Flexible parametric survival analysis using Stata:beyond the Cox model. College Station, Tex. U.S.A.: Stata Press; 2011. pp. 174-175.
14. Grønnesby JK, Borgan O. A method for Checking regression models in survival analysis based on the risk score. Lifetime Data Analysis 1996;2,315-328.
15. Gonen, M, Heller G. Concordance probability and discriminatory power in proportional hazards regression. Biometrika 2005;92:965–970.
16. Royston P, Sauerbrei W. A new measure of prognostic separation in survival data. Statistics Med 2004;23:723–748.
17. Vickers AJ, Van Calster B, Steyerberg EW. Net benefit approaches to the evaluation of prediction models, molecular markers, and diagnostic tests. BMJ. 2016 Jan 25;352.
18. Vickers AJ, Elkin EB. Decision curve analysis: a novel method for evaluating prediction models. Med Decis Making 2006;26(6):565–574.
19. Vickers AJ. Decision analysis for the evaluation of diagnostic tests, prediction models and molecular markers. Am Stat 2008;62:314–320.
20. Vickers AJ, Cronin AM, Elkin EB, Gonen M. Extensions to decision curve analysis, a novel method for evaluating diagnostic tests, prediction models and molecular markers. BMC Med Inform Decis Mak. 2008 Nov 26;8:53.
21. Van Calster B, Vickers AJ, Pencina MJ, Baker SG, Timmerman D, Steyerberg EW. Evaluation of markers and risk prediction models: Overview of relationships between NRI and decision-analytic measures. Med Decis Making 2013;33(4):490-501.
