# Supplementary figures and images for: Prognosis of severe acquired brain injury: Short and long-term outcome determinants and their potential clinical relevance after rehabilitation. A comprehensive approach to analyze cohort studies
Source: PLoS One. 2019 Sep 26;14(9):e0216507. doi: 10.1371/journal.pone.0216507 (PMC6762165; doi:10.1371/journal.pone.0216507)

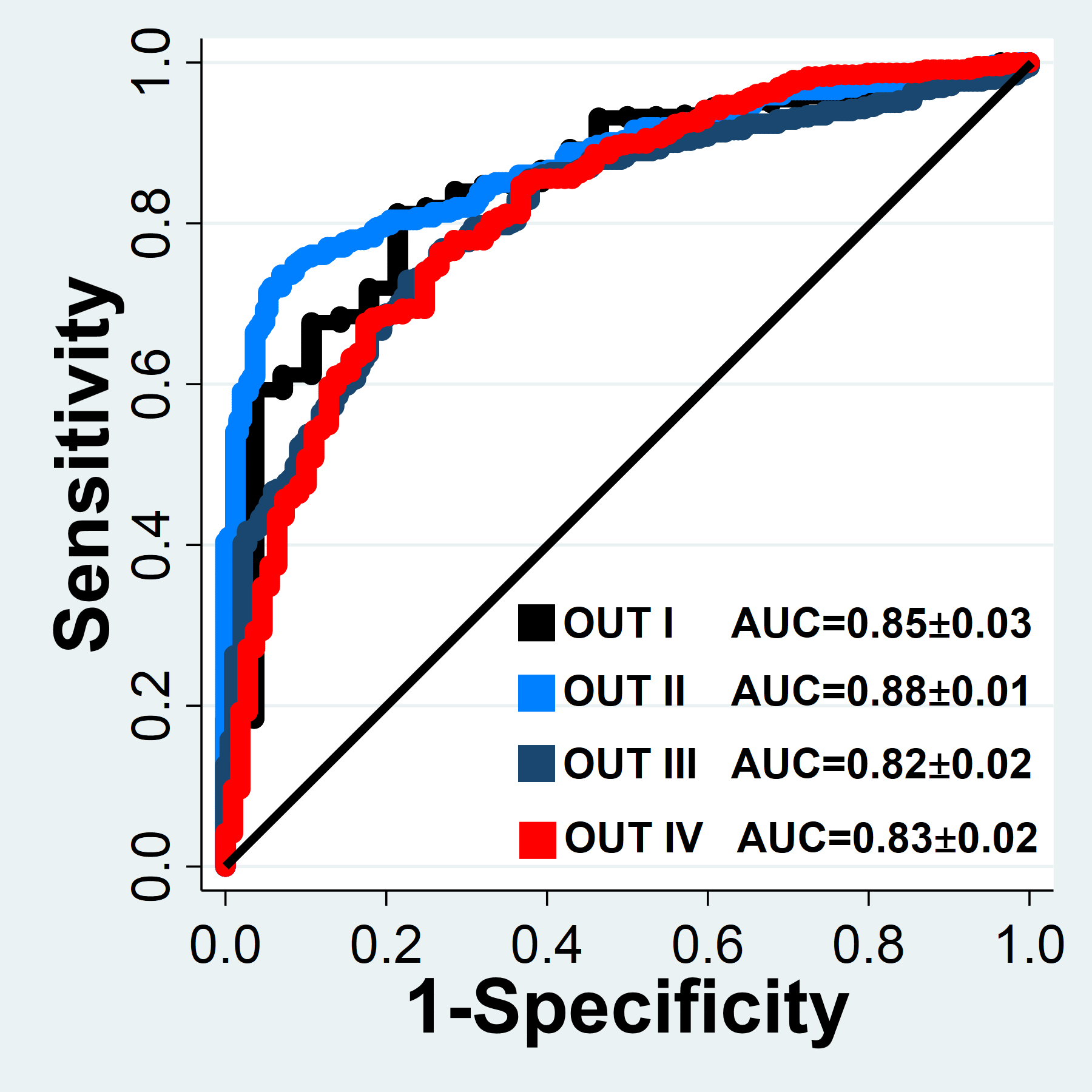

Supplement: S1 Fig — Jack-knife corrected receiver operating characteristics curves (ROC) relative to the four outcomes of the final GOLOGIT model. OUT I–IV: outcomes I–IV, AUC: area under the ROC curve. (TIF) [file pone.0216507.s007.tif]

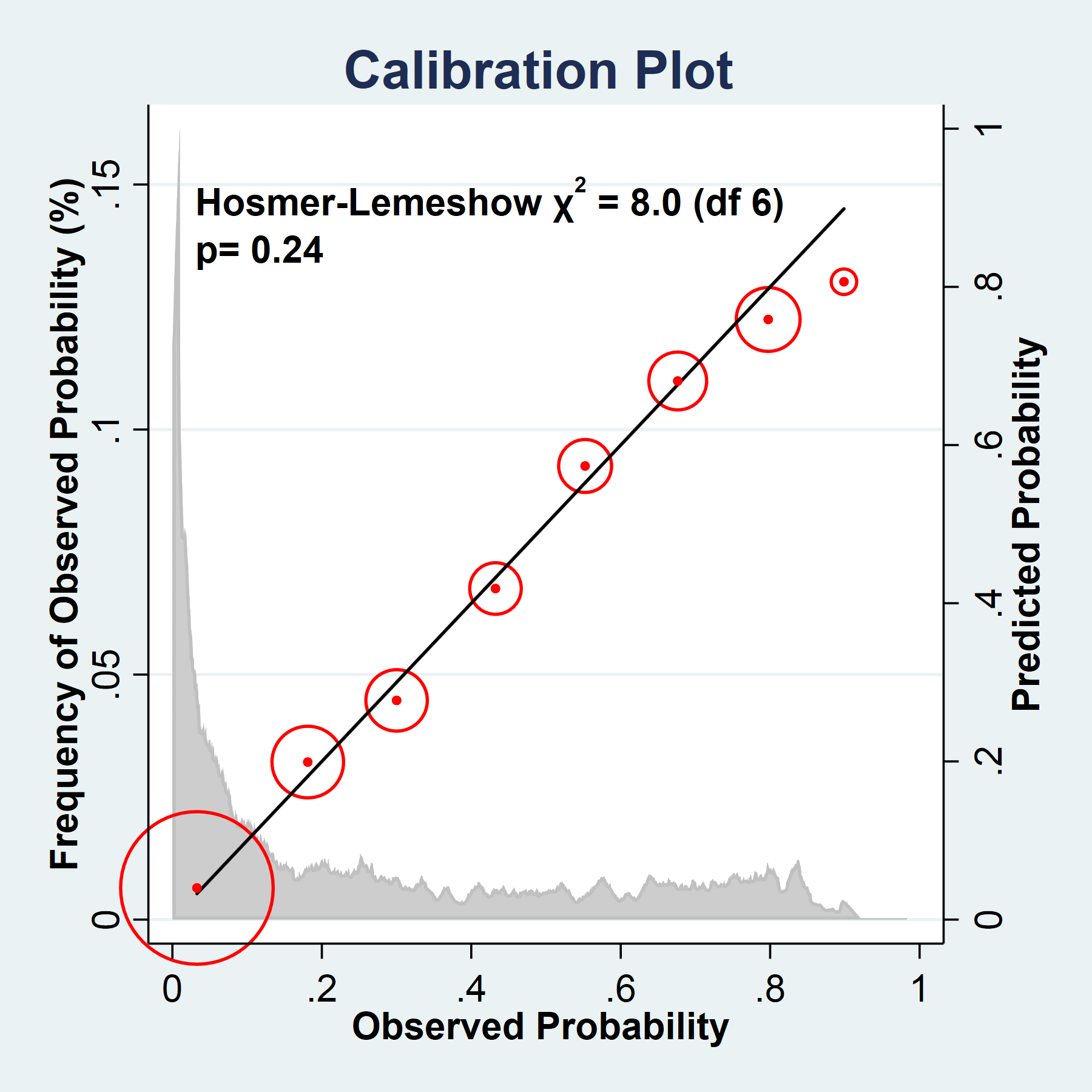

Supplement: S2 Fig — Jack-knife corrected calibration plot of observed probabilities vs predicted probabilities computed using jack-knife re-sampling procedure. Hollow circle diameters are proportional to group’s size. Grey shaded area outlines the frequency of the observed probabilities. (TIF) [file pone.0216507.s008.tif]
